# Hepatic Amyloidosis With Multiorgan Involvement

**DOI:** 10.14309/crj.0000000000000999

**Published:** 2023-04-19

**Authors:** Robert J. Duve, Tiberiu G. Moga, Kevin Yang, Thomas C. Mahl, Eric Dove

**Affiliations:** 1 Department of Internal Medicine, Jacobs School of Medicine and Biomedical Sciences, University at Buffalo, Buffalo, NY; 2 Division of Gastroenterology, Hepatology, and Nutrition, Jacobs School of Medicine and Biomedical Sciences, University at Buffalo, Buffalo, NY; 3 Jacobs School of Medicine and Biomedical Sciences, University at Buffalo, Buffalo, NY; 4 Department of Pathology and Anatomical Sciences, Jacobs School of Medicine and Biomedical Sciences, University at Buffalo, Buffalo, NY

**Keywords:** AL amyloid, ight chain amyloidosis, hepatic; light-chain, acute liver failure

## Abstract

Amyloidosis is a diverse entity that poses both diagnostic and treatment challenges. Whether systemic or local, amyloidosis has varied manifestations including occasional hepatic involvement. Hepatic amyloidosis, although rare, should be on the differential for those with unexplained hepatomegaly, cholestasis, alkaline phosphatase elevations, other associated organomegaly, and those with certain epidemiologic risks. In this study, we report a case of a man with systemic amyloid light chain amyloidosis with multiorgan involvement, acute liver injury, cholestasis, nephrotic syndrome, cardiomegaly, and bleeding diathesis.

## INTRODUCTION

Since amyloidosis was first described in 1853, the enlarged and dysfunctional organs that have resulted from the disorder have interested pathologists and clinicians alike. Amyloidosis is an umbrella term describing extracellular deposition of insoluble, misfolded proteins aggregating into β-pleated sheets with resultant organ dysfunction and architectural changes.^1^ More than 25 different amyloid proteins contribute to human disease, but the most common are amyloid light chain (AL), amyloid A, amyloid transport protein transthyretin, and dialysis-related amyloidosis.^2,3^ Amyloidosis can be acquired, as seen in AL amyloidosis with plasma cell dyscrasias or hereditary, seen in mutations with the TTR gene.^4^ Amyloidosis, particularly with cardiac involvement, is a significant risk of mortality. Amyloidosis-related mortality has increased from 1.77 to 3.96 per million between 1979 and 2015.^5^ Because of disease rarity and broad clinical manifestations, incidence is difficult to estimate accurately, but it is believed to be approximately 3–5 cases per million for the most common, AL, with hepatic involvement estimated in 9% of those.^6,7^ In 1 case series consisting of 80 patients with hepatic amyloidosis confirmed by liver biopsy, the most commonly seen clinical features were nephrotic syndrome, abnormal serum protein electrophoresis, and hepatomegaly disproportionate to level of liver enzyme abnormalities.^7^ Amyloidosis more commonly affects older persons, with a mean age of 63 years, males, and has higher mortality in African Americans.^8,9^

## CASE REPORT

A 55-year-old man presented for evaluation of shortness of breath, worsening over 2–3 weeks. This was accompanied by 2–3 months of progressive abdominal, genital, and lower extremity edema, preventing ambulation. Associated symptoms included productive cough and malaise. He denied fever, chills, chest pain, palpitations, abdominal pain, jaundice, pruritus, nausea, or vomiting. He had been noncompliant with his medications and was an active alcohol and tobacco user with last drink being 2 days before admission. Medical history included chronic lymphocytic leukemia (CLL), chronic obstructive pulmonary disease, polysubstance abuse, coronary artery disease s/p myocardial infarction, hypertension, type 2 diabetes mellitus, and deep vein thrombosis s/p inferior vena cava filter. His CLL was diagnosed 9 months earlier and was not being treated.

On presentation, he was hypoxemic requiring O_2_ by nasal cannula and in mild distress with wheezes heard throughout lung fields. There was extensive lower extremity edema and ascites. Complete blood count showed white blood count of 14.2, hemoglobin 14.5, platelets 450 (peaked at 507), and international normalized ratio (INR) 0.9. Comprehensive metabolic panel was significant for blood urea nitrogen 21, creatinine 2.1 (baseline), albumin 1.5, total bilirubin 1.5, alkaline phosphatase 2,229, aspartate transaminase 73, and alanine transaminase 27. B-type natriuretic peptide was 2,381. Urinalysis showed 3+ protein, dysmorphic red blood cells, and oval fat bodies. Computed tomography of the abdomen and pelvis without contrast revealed large volume abdominal ascites, diffuse anasarca, hepatomegaly with lobular hepatic contour, and unremarkable kidneys and heart. Echocardiogram was suggestive of amyloidosis, including findings of biventricular hypertrophy and global longitudinal strain. Abdominal ultrasound with Doppler showed hepatic steatosis, patent hepatic and portal vasculature, and low portal vein velocities 12–17 cm/s indicative of portal hypertension and splenomegaly. Renal ultrasound was unremarkable. Paracentesis was performed, showing serum‐ascites albumin gradient 1.2, total protein <0.5, and <250 polymorphonuclear cells.

**Figure 1.**
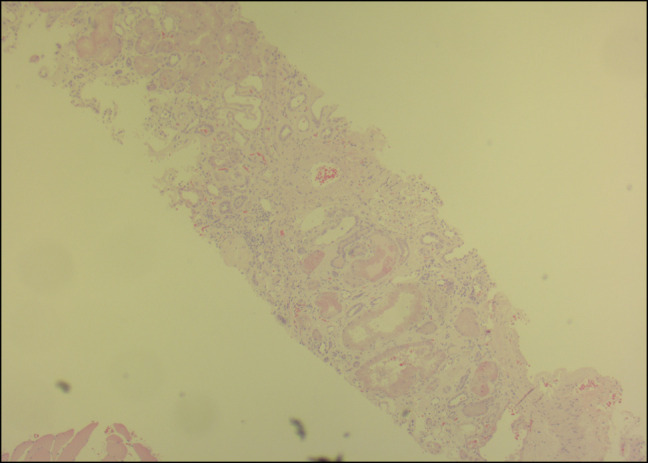
Hematoxylin and eosin section of renal biopsy specimen 40×.

**Figure 2.**
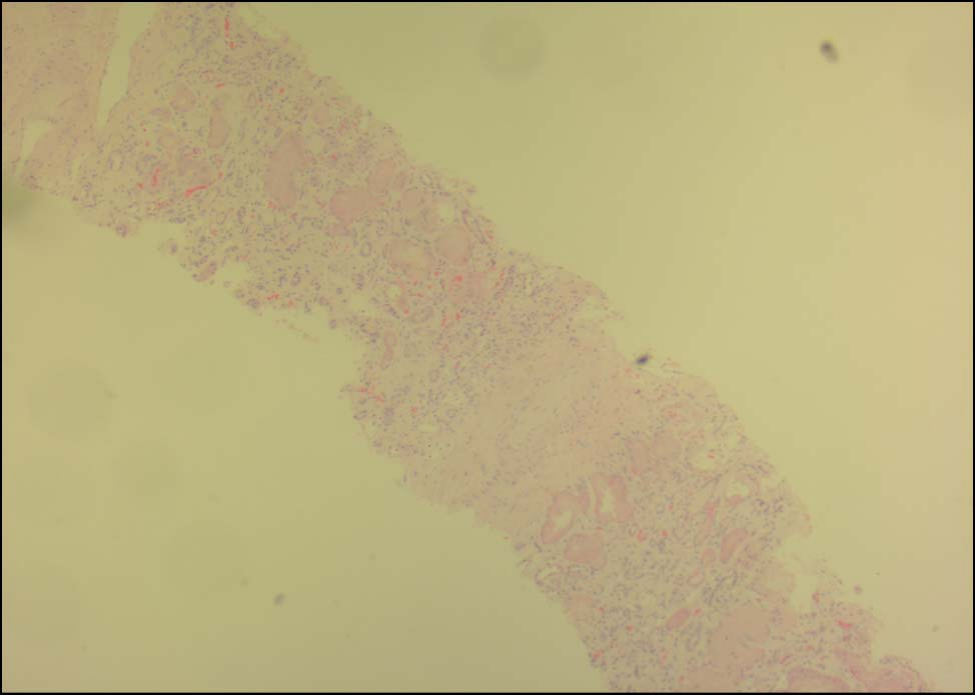
Hematoxylin and eosin section of renal biopsy specimen 40×.

**Figure 3.**
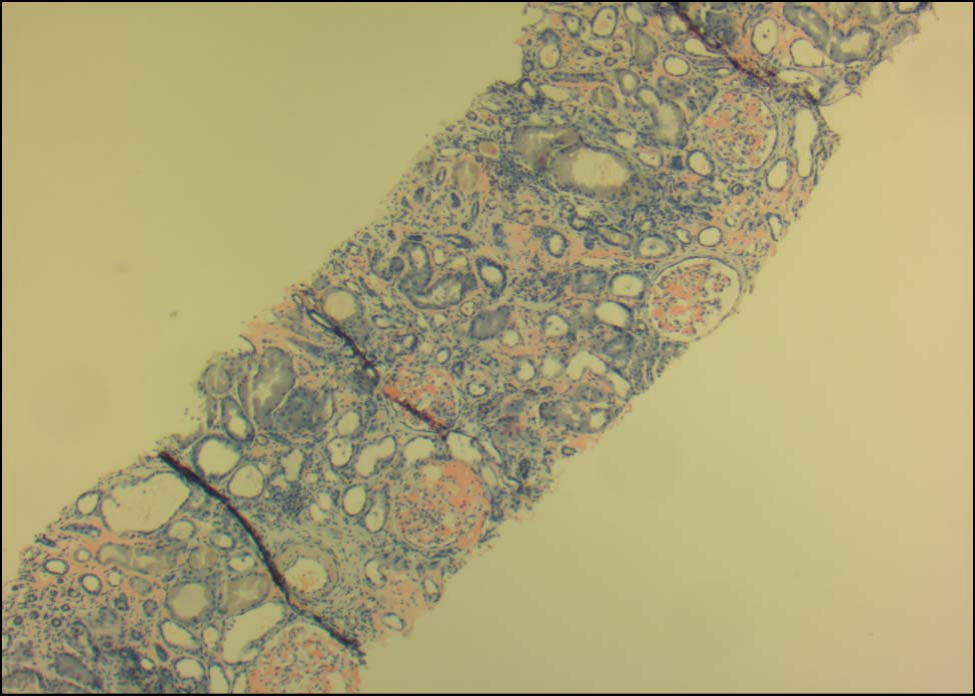
Renal biopsy specimen staining positive for Congo Red 100×.

**Figure 4.**
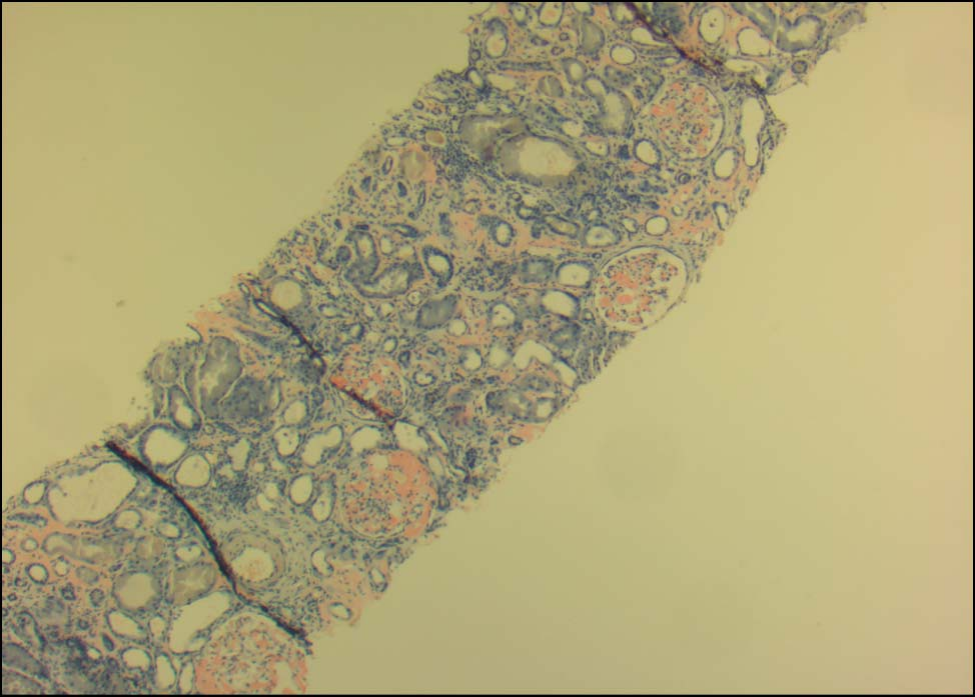
Renal biopsy specimen staining positive for Congo Red 100×.

Treatment was started for acute mixed respiratory failure secondary to heart failure with preserved ejection fraction and chronic obstructive pulmonary disease. Serum protein electrophoresis, urine protein electrophoresis, and free light-chain assay were unremarkable. Creatinine rose to 4.0, and he had severe proteinuria (>10 g/d). Renal biopsy was positive for AL on Congo Red staining (Figures 1–4), confirming nephrotic syndrome because of amyloidosis. Bone marrow biopsy did not show monoclonality but did show lymphocytosis and stained positive for amyloid.

Renal function continued to deteriorate despite aggressive medical therapy. Dialysis was recommended, but patient had refused. His liver chemistries worsened, and ursodeoxycholic acid was started for cholestasis. Liver biopsy was deferred because of coagulopathy and risk of liver fracture. He developed encephalopathy with possible disseminated intravascular coagulation. Final chemistries were blood urea nitrogen 158, creatinine 10.8, alkaline phosphatase 2,613, aspartate transaminase 206, alanine transaminase 82, total bilirubin 7.6, albumin 1.6, and INR 2.1. He ultimately transitioned to comfort measures and was discharged to hospice care. He expired within 1 month of discharge.

## DISCUSSION

The criteria for acute liver failure are impaired synthetic function with INR >1.5 and encephalopathy for fewer than 26 weeks without pre-existing chronic liver disease.^10^ The differential is broad and includes viral hepatitis, autoimmune, metabolic, drug-induced, and idiopathic causes.^11^ The patient in our case met all the above criteria for acute liver failure. Although we did not obtain liver biopsy, it is likely attributable to hepatic amyloidosis, given the associated positive kidney biopsy for AL amyloid.^7^ To make an initial diagnosis of hepatic amyloidosis, it would have taken a high degree of suspicion early in the hospital course because of the patient's rapid multiorgan dysfunction. The risk of bleeding was deemed too high because of patient's bleeding diathesis, most likely contributed by coagulopathy of liver disease with INR 2.1 and the predilection for factor X to bind amyloid fibrils.^12^ Liver biopsy was deferred because of coagulopathy and also risk of liver fracture in the setting of amyloidosis. Furthermore, the development of the patient's AL amyloid is likely secondary because of his untreated CLL because this is a rare but documented cause.^13^

Symptoms of hepatic amyloidosis can include hepatomegaly, weight loss, fatigue, abdominal discomfort, and anorexia.^14^ The most common examination and laboratory findings are hepatomegaly and mild elevations in alkaline phosphatase.^15^ Complications that were commonly seen in 1 case series included abnormal liver function tests, portal hypertension, hepatic failure, associated nephrotic syndrome, hepatomegaly, and congestive heart failure.^16^ An electrocardiogram abnormality that is often seen with cardiac involvement is fascicular block because of preferential infiltration of the His-Purkinje system.^17^ Another case series that followed 98 patients with amyloidosis that had biopsy-proven liver involvement found that median survival was 8.5 months. Independent risk factors that were associated with decreased survival were heart failure, elevated bilirubin, and platelets >500.^18^ Our patient had all of these features. In addition, our patient's continual third spacing was likely contributed to by hypoalbuminemia and portal hypertension contributed to by associated nephrotic syndrome and hepatic involvement. Other causes of portal hypertension that were ruled out include veno-occlusive disease and right heart failure because he did not have hematopoietic stem cell transplant, patent hepatic vasculature ruling-out Budd-Chiari, and no significant right heart failure was seen on echo.

In our case, the patient presented with the majority of symptoms and physical examination findings that are seen in systemic amyloidosis with hepatic involvement. Manifestations seen included nephrotic syndrome, acute liver failure, and evidence of cardiac infiltration. These findings in combination with untreated CLL and a kidney biopsy positive for AL amyloid with Congo Red staining support a diagnosis of hepatic amyloidosis secondary to untreated malignancy.

## DISCLOSURES

Author contributions: R. Duve and T. Moga wrote and edited the article and reviewed the literature. K. Yang contributed to writing the article. E. Dove reviewed and provided the pathology slides. T. Mahl edited the article and is the article guarantor. All authors approved the final version of the manuscript.

Financial disclosure: None to report.

Informed consent was obtained for this case study.
